# The prognostic value of ^18^F-FDG PET/CT intra-tumoural metabolic heterogeneity in pretreatment neuroblastoma patients

**DOI:** 10.1186/s40644-022-00472-4

**Published:** 2022-07-05

**Authors:** Jun Liu, Yukun Si, Ziang Zhou, Xu Yang, Cuicui Li, Luodan Qian, Li Juan Feng, Mingyu Zhang, Shu Xin Zhang, Jie Liu, Ying Kan, Jianhua Gong, Jigang Yang

**Affiliations:** 1 Department of Nuclear Medicine, Beijing Friendship Hospital, Capital Medical University, Beijing, China; 2 Oncology Department, Institute of Medicinal Biotechnology, Chinese Academy of Medical Sciences & Peking Union Medical College, Beijing, China

**Keywords:** Neuroblastoma, ^18^F-FDG FDG PET/CT, Intra-tumoural metabolic heterogeneity, Prognosis

## Abstract

**Background:**

Neuroblastoma (NB) is the most common tumour in children younger than 5 years old and notable for highly heterogeneous. Our aim was to quantify the intra-tumoural metabolic heterogeneity of primary tumour lesions by using ^18^F-FDG PET/CT and evaluate the prognostic value of intra-tumoural metabolic heterogeneity in NB patients.

**Methods:**

We retrospectively enrolled 38 pretreatment NB patients in our study. ^18^F-FDG PET/CT images were reviewed and analyzed using 3D slicer software. The semi-quantitative metabolic parameters of primary tumour were measured, including the maximum standard uptake value (SUVmax), metabolic tumour volume (MTV), and total lesion glycolysis (TLG). The areas under the curve of cumulative SUV-volume histogram index (AUC-CSH index) was used to quantify intra-tumoural metabolic heterogeneity. The median follow-up was 21.3 months (range 3.6 - 33.4 months). The outcome endpoint was event-free survival (EFS), including progression-free survival and overall survival. Survival analysis was performed using Cox regression models and Kaplan Meier survival plots.

**Results:**

In all 38 newly diagnosed NB patients, 2 patients died, and 17 patients experienced a relapse. The AUC-CSH_total_ (r=0.630, P<0.001) showed moderate correlation with the AUC-CSH_40%_. In univariate analysis, chromosome 11q deletion (P=0.033), Children's Oncology Group (COG) risk grouping (P=0.009), bone marrow involvement (BMI, P=0.015), and AUC-CSH_total_ (P=0.007) were associated with EFS. The AUC-CSH_total_ (P=0.036) and BMI (P=0.045) remained significant in multivariate analysis. The Kaplan Meier survival analyses demonstrated that patients with higher intra-tumoural metabolic heterogeneity and BMI had worse outcomes (log-rank P=0.002).

**Conclusion:**

The intra-tumoural metabolic heterogeneity of primary lesions in NB was an independent prognostic factor for EFS. The combined predictive effect of intra-tumoural metabolic heterogeneity and BMI provided prognostic survival information in NB patients.

## Background

Neuroblastoma (NB) is the most common tumour in children younger than 5 years old and accounts for approximately 15% of all paediatric cancer deaths [1, 2]. NB originates from multipotent neural crest cells and could occur anywhere along the sympathetic chain [3]. NB is notable for highly heterogeneous clinical symptoms and outcomes, ranging from total regression to incurable multi-foci- and multi-drug-resistant disease [2, 4]. Although great progress has been made in treating NB, the outcome for high-risk patients remains poor, nearly 50% patients without long-term survival [4, 5]. Most NB patients with the aggressive disease do not show sustained responses to anticancer therapy, thus further emphasize the importance of comprehensive evaluation of the heterogeneity of tumours and individualized treatment of patients [2, 6].

^18^F-FDG PET/CT has been widely used in diagnosing and prognostic evaluation of various tumours. ^18^F-FDG PET/CT metabolic parameters have been used as biomarkers to predict outcomes for cancer patients [7]. Acquiring more risk factors at the time of diagnosis could allow tailor treatment for each patient individually. For example, the maximum standard uptake value (SUVmax), the metabolic tumour volume (MTV), and the total lesion glycolysis (TLG) were significantly correlated with higher rates of recurrence and progression-free survival in many cancers [8–11]. While, some limitations of SUV-derived measurements still exist, as the tracer uptake usually is not homogeneously distributed throughout the tumour [7]. The intra-tumoural metabolic heterogeneity is defined as different tumour cells showing specific phenotypic and morphologic features in tumours [12]. It is reported that tumours with higher heterogeneity might have a worse prognosis [7]. A variety of imaging parameters are available to assess tumour heterogeneity. Recently, there has been increasing interest in assessing intra-tumoural metabolic heterogeneity by using ^18^F-FDG PET/CT. Some studies have reported that intra-tumoural metabolic heterogeneity is correlated with treatment failure, the higher possibility of metastasis, and poor prognosis in various tumours, such as lung cancer, breast cancer and cervical cancer [7, 13–15]. However, no existing study has explored the prognostic value of intra-tumoural metabolic heterogeneity on ^18^F-FDG PET/CT in paediatric NB patients.

The present study aimed to explore the prognostic value of ^18^F-FDG PET/CT metabolic imaging parameters and intra-tumoural metabolic heterogeneity in NB patients.

## Materials and Methods

### Patients

We retrospectively searched our database to identify all patients with NB who were performed ^18^F-FDG PET/CT between January 2018 to December 2019. Newly diagnosed paediatric patients with histopathologic confirmed of NB on baseline ^18^F-FDG PET/CT scans before treatment were included in the study. Patients with alternative histopathologic findings, or receiving treatment before ^18^F-FDG PET/CT scan, or without a baseline examination were excluded. Thirty-eight patients who met our criteria were included in this study. We would record the date of death or disease progression or the last day of follow-up through patient medical charts or telephone calls. Median follow-up was 21.3 months (range 3.6 - 33.4 months). The endpoint was event-free survival (EFS), including progression-free survival and overall survival. This retrospective study was approved by our Institutional Review Board and the requirement of informed consent was waived.

### PET/CT Scan Parameters

All patients were performed PET/CT scans (Siemens Biograph MCT, Germany) following manufacturer's recommended clinical protocol. After fasting for at least 4 h and blood glucose level lower than 200 mg/dL, patients would be injected ^18^F-FDG (3.7 MBq/kg). Patients were performed whole-body PET scan with low-dose CT imaging from the skull to the upper part of the thigh without contrast medium after approximately 1 h after ^18^F-FDG intravenous injection. Sedation would be considered if necessary. According to our clinical protocol, PET images were reconstructed using the ordered subset expectation maximization algorithm with a gaussian filter. CT images were acquired according to the following parameters: slice thickness, 5 mm; tube voltage, 120 kV; tube current, 200 mAs; and FOV, 70 cm^2^. CT images were used for attenuation correction and anatomical co-registration.

### PET/CT Image Analysis

PET/CT images were reviewed using 3D slicer software (version: 4.13.0), a free and open-source software widely used by physicians and researchers [16]. Axial, coronal, and sagittal PET images, CT images, and fused PET/CT images were reviewed by two experienced nuclear medicine physicians to identify the primary NB lesion. All primary NB lesions were manually drawn by nuclear medicine physician. The semi-quantitative PET/CT parameters including the SUVmax, the mean standardized uptake value (SUVmean), the peak standardized uptake value (SUVpeak calculated using an automated computed maximal average SUV in 1.0 cm^3^ spherical volume within the tumour) were measured. The MTV was calculated by two methods outlining the primary tumour for total-lesion (MTV_total_) and 40% SUVmax-threshold (MTV_40%_). The TLG was calculated by two methods TLG_total_ (MTV_total_ × SUVmean) and TLG_40%_ (MTV_40%_ × SUVmean).

**Fig. 1:**
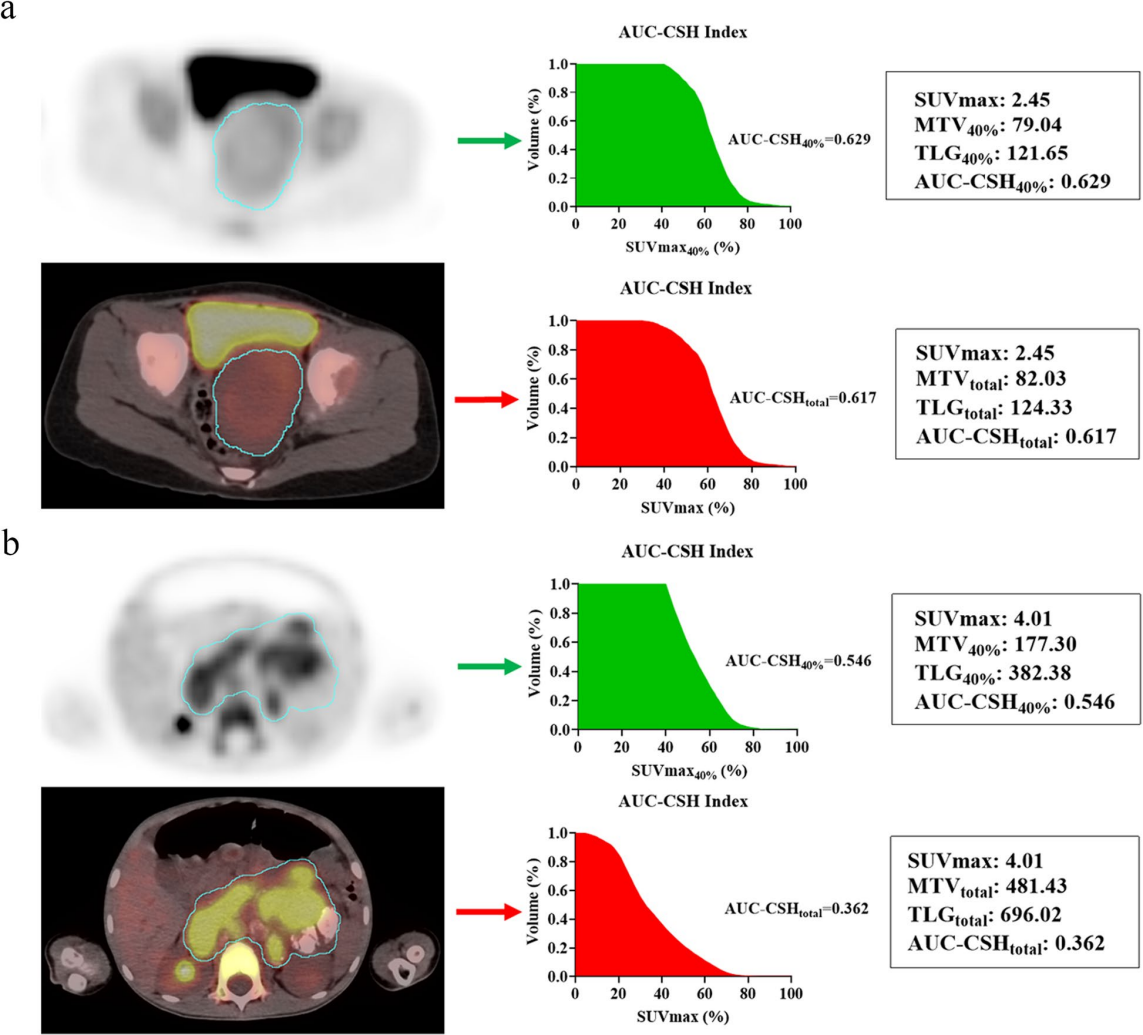
AUC-CSH index and metabolic parameters of two neuroblastoma patients **a** Homogeneous neuroblastoma had MTV_40%_ of 79.04, TLG_40%_ of 121.65 and AUC-CSH_40%_ of 0.629, and relative stable MTV_total_ of 82.03, TLG_total_ of 124.33, and AUC-CSH_total_ of 0.617. **b** Heterogeneous neuroblastoma had MTV_40%_ of 177.30, TLG_40%_ of 382.38 and AUC-CSH_40%_ of 0.546, and significant different MTV_total_ of 481.43, TLG_total_ of 696.02, and AUC-CSH_total_ of 0.362

The areas under the curve of cumulative SUV-volume histogram index (AUC-CSH index) were considered a novel method to characterize intra-tumoural metabolic heterogeneity [14, 17]. The AUC-CSH index was calculated as the percent volume greater than the percentage of SUVmax, and a lower AUC-CSH index corresponds to a more heterogeneous distribution. The AUC-CSH index was calculated by two segmentation methods outlining the primary tumour for total-lesion (AUC-CSH_total_) and 40% SUVmax-threshold (AUC-CSH_40%_) (Fig. 1).

### Statistical Analysis

Continuous data were presented as mean ± standard deviation (mean ± SD) for normal or median and interquartile ranges for non-normal distribution. Categorical data were presented as numbers (percentages). The clinical data of patients between different groups were analyzed using the Mann-Whitney U test and Chi-square test. The differences between AUC-CSH_total_ and AUC-CSH_40%_ in different group patients were examined using T-test and Wilcoxon signed rank test, and the correlations between them were examined using Spearman correlation coefficients. The Cox regression models and Kaplan Meier survival curves were used to identify the prognostic factors for EFS. All analyses were performed with SPSS 22.0 software (IBM, Incorporation, Chicago, IL, USA). A two-tail *P* <0.05 was considered statistically significant.

## Results

### Patients' Characteristics

A total of 38 patients with pathology-proven newly diagnosed NB were included in this retrospective study. The age (mean ± SD) of those patients was 3.64 ± 2.17 years. Based on the patient's primary lesion location, 34 patients had lesions located in the abdomen and 4 patients had lesions located in pelvic cavity. There were 2 patients for International Neuroblastoma Staging System (INSS) stage I, one patient for stage II, 6 patients for stage III, and 29 patients for stage IV. According to the Children's Oncology Group (COG) risk grouping, 2 patients were low risk, 14 patients were intermediate risk and 22 patients were high risk. There was a significant difference in COG risk grouping between the event-free group and the event group (Details were summarized in Table 1). In terms of treatment, 35 patients received neoadjuvant chemotherapy, all patients underwent surgery and 37 patients received post-operative chemotherapy. During the follow-up period, 2 patients died and 17 patients experienced a relapse (Details were summarized in Table 2).

**Table 1:** Patients’ characteristics

Patient Characteristics	Total	Without event	With event	P
Age (years)	3.636±2.166	3.264±2.131	4.008±2.194	0.402
Gender				
Male	14(36.8%)	5(26.3%)	9(47.4%)	0.313
Female	24(63.2%)	14(73.7%)	10(52.6%)	
Tumor primary site				
Abdomen	34(89.5%)	16(84.2%)	18(94.7%)	0.604
Non-Abdomen	4(10.5%)	3(15.8%)	1(5.3%)	
INSS				
Stage 1	2(5.3%)	2(10.5%)	0(0.0%)	0.178
Stage2	1(2.6%)	1(5.3%)	0(0.0%)	
Stage3	6(15.8%)	4(21.1%)	2(10.5%)	
Stage4	29(76.3%)	12(63.1%)	17(89.5%)	
COG				
Low risk	2(5.3%)	2(10.5%)	0(0.0%)	0.027*
medium risk	14(36.8%)	10(52.6%)	4(21.1%)	
high risk	22(57.9%)	7(36.9%)	15(78.9%)	

*INSS* International Neuroblastoma Staging System, *COG* Children's Oncology Group, *P < 0.05

**Table 2:** Patients’ treatments and follow-up

Clinical Characteristics		Patients
Neoadjuvant chemotherapy	Yes	35 (92.1%)
	No	3 (7.9%)
Surgery	Yes	38 (100%)
	No	0 (0%)
Post-operative chemotherapy	Yes	37 (97.4%)
	No	1 (2.6%)
Endpoint events	Dead	2 (5.3%)
	Recurrence or progression	17 (44.7%)
	Disease free	19 (50.0%)

### Overall comparison two segmentation methods

We comprehensively compared MTV_total_, TLG_total_ and AUC-CSH_total_ with MTV_40%_, TLG_40%_ and AUC-CSH_40%_. In the test of variance, MTV_total_ and MTV_40%_ (*P*<0.001), TLG_total_ and TLG_40%_ (*P*<0.001), AUC-CSH_total_ and AUC-CSH_40%_ (*P*<0.001) were all significantly different. In the correlation analysis, MTV_total_ and MTV_40%_ (*r*=0.821, *P*<0.001), TLG_total_ and TLG_40%_ (*r*=0.935, *P*<0.001) showed excellent correlation, AUC-CSH_total_ and AUC-CSH_40%_ (*r*=0.630, *P*<0.001) only showed moderate correlation compared to MTV and TLG. More information was generalized in Table 3.

**Table 3:** Comparison of two segmentation methods

Variable		Value	Z	P	r	P
MTV			-5.303	<0.001*	0.821	<0.001*
	MTV_40%_	121.62 (52.95, 220.37)				
	MTV_total_	236.35 (132.94, 354.86)				
TLG			-5.303	<0.001*	0.935	<0.001*
	TLG_40%_	304.89 (133.32, 517.74)				
	TLG_total_	442.85 (237.40, 713.71)				
AUC-CSH			-4.971	<0.001*	0.630	<0.001*
	AUC-CSH_40%_	0.561±0.481				
	AUC-CSH_total_	0.463±0.098				

*MTV* metabolic tumour volume, *TLG* total lesion glycolysis, *AUC-CSH* areas under the curve of cumulative SUV-volume histogram, *P < 0.05

To explore the effect of intra-tumoural metabolic heterogeneity parameters in two segmentation methods, we further compared AUC-CSH_total_ and AUC-CSH_40%_ between different subgroups of patients. AUC-CSH_total_ (*P*=0.018) had a significant difference between the high-risk group and the low-medium-risk group, while AUC-CSH_40%_ (*P*=0.965) showed no significant difference. Similarly, AUC-CSH_total_ also showed significant difference in bone marrow involvement (BMI) group (*P*=0.03) and event-free group (*P*<0.001). AUC-CSH_40%_ only showed a significant difference in the event-free group (*P*=0.009). Both AUC-CSH_total_ (*P*=0.961) and AUC-CSH_40%_ (*P*=0.947) showed no significant difference in myelocytomatosis viral oncogene neuroblastoma-derived homolog (MYCN) normal group compared to MYCN acquired and amplified group. All details were summarized in Table 4.

**Table 4:** Comparison of AUC-CSH_total_ and AUC-CSH_40%_ between different subgroups

Variable		AUC-CSH_40%_	P	AUC-CSH_total_	P
COG			0.965		0.018*
	Low and medium risk	0.560±0.060		0.506±0.108	
	High risk	0.561±0.039		0.431±0.079	
BMI					
	No	0.563±0.070	0.914	0.516±0.113	0.030*
	Yes	0.560±0.038		0.441±0.007	
MYCN					
	Normal	0.560±0.054	0.947	0.465±0.113	0.861
	Acquired and amplify	0.561±0.039		0.459±0.077	
EFS					
	Without event	0.581±0.049	0.009*	0.515±0.101	<0.001*
	With event	0.541±0.039		0.410±0.062	

*COG* Children's Oncology Group, *BMI* bone morrow involvement, *MYCN* myelocytomatosis viral oncogene neuroblastoma derived homolog, *EFS* event-free survival, *AUC-CSH* areas under the curve of cumulative SUV-volume histogram, *P < 0.05

### Survival analysis

**Fig. 2:**
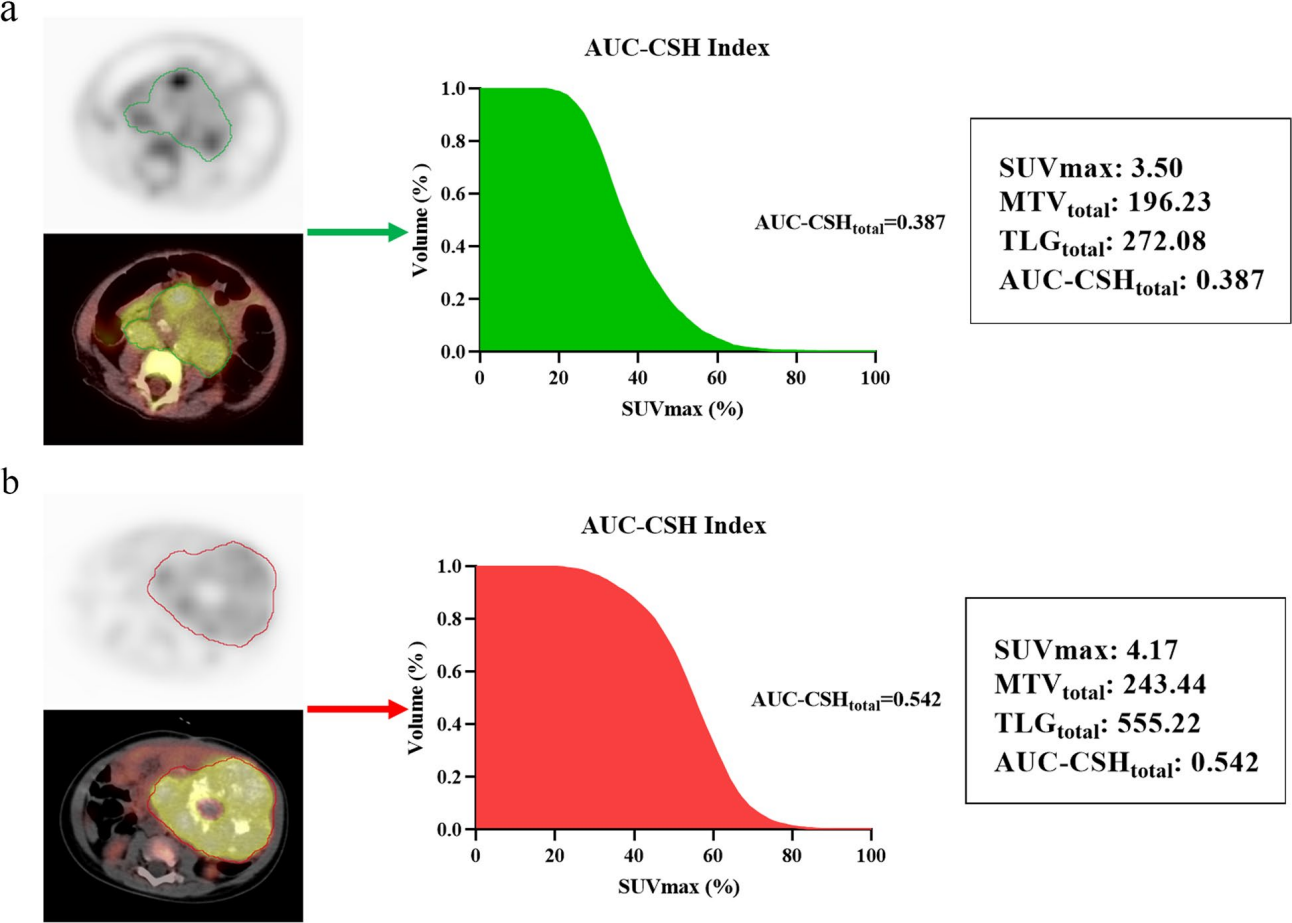
Compared of two neuroblastoma patients with different AUC-CSH index and metabolic parameters. **a** 1-year-old girl with INSS 4 stage neuroblastoma had SUVmax of 3.5, MTV_total_ of 196.23, TLG_total_ of 272.08, and a lower AUC-CSH_total_ of 0.387. The patient was disease progression at 3.6 months and dead at 6.2 months, after ^18^F-FDG PET/CT examination. **b** 1-year-old boy with INSS 3 stage neuroblastoma had SUVmax of 4.17, MTV_total_ of 243.44, TLG_total_ of 555.22, and a higher AUC-CSH_total_ of 0.542. The patient was disease free at 32.5 months after ^18^F-FDG PET/CT examination

In univariate analysis that some clinical characteristics, examination results and semi-quantitative metabolic parameters were included. Univariate analysis demonstrated that chromosome 11q deletion (*P*=0.033), COG risk group (*p*=0.009), BMI (*P*=0.015), AUC-CSH_total_ (*P*=0.007) were associated with EFS. Most semi-quantitative PET/CT metabolic parameters including SUVmean (*P*=0.052), SUVmax (*P*=0.237), SUVpeak (*P*=0.358), MTV_total_ (*P*=0.615), MTV_40%_(*P*=0.9), AUC-CSH_40%_ (*P*= 0.052) were not statistically significant (Fig. 2).

Variables with statistical significance would be included in multivariate Cox proportional hazards regression analysis with backward stepwise. AUC-CSH_total_ (HR: 0.005, 95%CI:0.000-0.705, *P*=0.036) and BMI (HR: 4.677, 95%CI:1.032-21.205, *P*=0.045) remained to be significant in multivariate analysis. While chromosome 11q deletion (*P*=0.359) and COG risk group (*P*=0.125) showed no significance in the multivariate analysis. Results were generalized in Table 5.

**Table 5:** Univariate and multivariate analysis for event-free survival

Survival analysis				
Variable	Univariate		Multivariate	
	HR (95 % CI)	P	HR (95 % CI)	P
Age	1.106 (0.895-1.367)	0.351		
Tumor primary site	0.337(0.045-2.533)	0.291		
MYCN	1.492(0.605-3.680)	0.385		
1p	1.202(0.794-1.820)	0.384		
11q	1.529(1.035-2.259)	0.033*		0.359
INSS stage	3.508(0.922-13.551)	0.066		
COG group	4.719(1.474-15.113)	0.009*		0.125
NSE	1.000(1.000-1.001)	0.256		
LDH	1.000(1.000-1.001)	0.336		
PHOX2B	1.000(1.000-1.001)	0.212		
BMI	6.336(1.426-28.153)	0.015*	4.677(1.032-21.205)	0.045*
SUVmean	0.981(0.644-1.493)	0.928		
SUVmax	1.083(0.949-1.237)	0.237		
SUVpeak	1.085(0.912-1.290)	0.358		
MTV_total_	1.000(0.999-1.002)	0.615		
MTV_40%_	1.000(0.997-1.003)	0.9		
TLG_total_	1.000(0.999-1.001)	0.918		
TLG_40%_	1.000(0.999-1.001)	0.804		
AUC-CSH_total_	0.001(0.001-0.164)	0.007*	0.005(0.000-0.705)	0.036*
AUC-CSH_40%_	0.001(0.001-1.103)	0.052		

*MYCN* myelocytomatosis viral oncogene neuroblastoma derived homolog, *INSS* International Neuroblastoma Staging System, *COG* Children's Oncology Group, *NSE* neuron-specific enolase, *LDH* lactate dehydrogenase, *PHOX2B* paired-like homebox 2B, *BMI* bone morrow involvement, *SUVmean* the mean standardized uptake value, *SUVmax* the maximum standard uptake value, *SUVpeak* the peak standardized uptake value, *MTV* metabolic tumour volume, *TLG* total lesion glycolysis, *AUC-CSH* areas under the curve of cumulative SUV-volume histogram, *HR*: hazard rate, *CI* confidence interval, *P < 0.05

### Effect of AUC-CSHtotal and BMI on survival

**Fig. 3:**
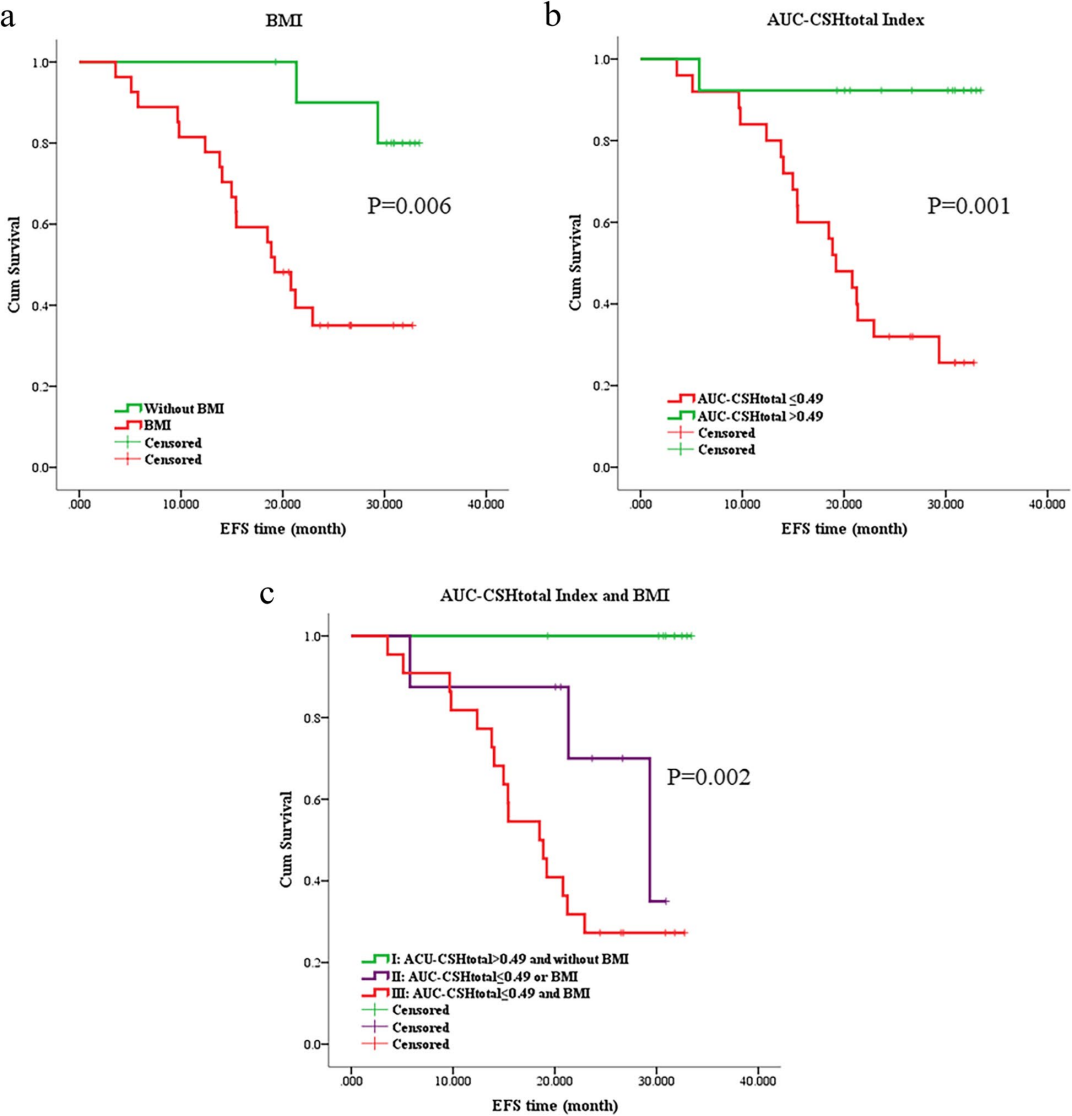
Kaplan-Meier survival curves. **a** Survival curves for BMI (*P*=0.06); **b** Survival curves based on optimum cut-off value for AUC-CSH_total_ index (*P*=0.001); **c** Survival curves for the combined predictive effect of AUC-CSH_total_ index and BMI (*P*=0.002)

We further investigated the combined predictive effect of AUC-CSH_total_ and BMI. The optimum cut-off value of AUC-CSH_total_ was 0.49, dichotomized by receiver operating characteristics (ROC) analysis. All patients were stratified into three groups based on the optimum cut-off value of AUC-CSH_total_ and BMI. Group I included patients with AUC-CSH_total_ > 0.49 and without BMI; Group II included patients with AUC-CSH_total_ ≤ 0.49 or BMI; group III included patients who had both AUC-CSH_total_ ≤ 0.49 and BMI. As expected, the Kaplan Meier survival analyses demonstrated that patients with AUC-CSH_total_ ≤ 0.49 and BMI had worse outcomes (log-rank *P*=0.002) (Figure 3).

## Discussion

The present study aimed to determine the prognostic value of semi-quantitative PET/CT-derived parameters and intra-tumoural metabolic heterogeneity in pretreated NB patients. Our research revealed that intra-tumoural metabolic heterogeneity quantified by AUC-CSH index could perfectly predict EFS. The survival analysis showed that the AUC-CSH_total_ index was an independent predictor, and those patients with lower AUC-CSH_total_ indexes (higher intra-tumoural heterogeneity) had worse outcomes.

Intra-tumoural heterogeneity is caused by the cumulative effects of many related genes [18]. Intra-tumoural heterogeneity is defined as different subgroups of tumour cells types within an individual tumour that often lead to treatment failure and drug resistance [19, 20]. There is a growing interest in analyzing intra-tumoural heterogeneity using different ^18^F-FDG PET/CT metabolic parameters. The intra-tumoural heterogeneity expression by ^18^F-FDG PET/CT quantitative metabolic parameters is called intra-tumoural metabolic heterogeneity index. Several studies have used the^18^F-FDG PET/CT intra-tumoural metabolic heterogeneity index to predict the prognosis of various cancers [21–23]. Esther et al. evaluated the prognostic significance of intra-tumoural metabolic heterogeneity in HPV-positive primary oropharyngeal squamous cell carcinoma, and demonstrated that the AUC-CSH index was an independent prognostic factor for EFS [23]. Similarly, Daniella et al. also explored the utility of ^18^F-FDG PET/CT intra-tumoural metabolic heterogeneity in cervical cancer patients and ^18^F-FDG PET/CT intra-tumoural metabolic heterogeneity could significantly predict progression-free survival and overall survival [7]. NB is a heterogeneous tumour with highly heterogeneous clinical symptoms and outcomes. However, no studies have explored the association between ^18^F-FDG PET/CT intra-tumoural metabolic heterogeneity and NB patient outcomes. Our study firstly explored the role of ^18^F-FDG PET/CT intra-tumoural metabolic heterogeneity in predicting the EFS in the highly heterogeneous NB.

Malignant tumours manifest heterogeneity in many respects, not only in the gene differential expression and biological composition but also in behavioural and metabolic characteristics. Within the same tumour, even at the same stage, significant heterogeneity is also existed, such as different growth rates, vascularity and necrosis of the same tumour cell population [24]. FDG uptake is not usually homogeneously across the tumour which is influenced by several factors, including tumour necrosis, cellular proliferation, and hypoxia [25]. Currently, there are many methods to evaluate intra-tumoural heterogeneity by using ^18^F-FDG PET/CT metabolic parameters, such as coefficient of variance (COV) [26], cumulative SUV-volume histogram (CSH) [27], AUC-CSH index [14], texture analysis [28], heterogeneity factors [29] and fractal analysis [25, 30]. The AUC-CSH index is a widely accepted method to assess intra-tumoural heterogeneity and is used for differential diagnosis and prognostic assessment [17, 23, 31]. There are two different methods for calculating AUC-CSH index, including total-lesion based and SUVmax-threshold based. We compared the AUC-CSH index between the two methods and found only a moderate correlation. Esther Mena's study also found a moderate correlation about the AUC-CSH index in pancreatic adenocarcinomas [32]. While we further compared the AUC-CSH index in different subgroups of NB patients, which differed from previous studies [7, 23]. The AUC-CSH_40%_ index only showed significant differences between the event group and the event-free group. The AUC-CSH_total_ index showed significantly different in more groups of patients, compared to AUC-CSH_40%_. The AUC-CSH_total_ index contained more hypometabolic regions, which were also parts of the tumours. For heterogeneous NB, which contained calcification and necrosis, the AUC-CSH_total_ could be as a more comprehensive representation of intra-tumoural heterogeneity of NB compared to AUC-CSH_40%_.

In the current study, we had investigated the prognostic value of semi-quantitative PET/CT-derived parameters and clinicopathological factors in NB patients. Interestingly, traditional semi-quantitative metabolic parameters in our study did not show significant association with EFS, such as MTV and TLG. Similarly, Sung AJ et al. evaluated the prognostic significance of ^18^F-FDG PET/CT in NB patients, indicating that MTV and TLG were not prognostic factors [33]. Lee et al. explored the utility of pretreatment ^18^F-FDG PET/CT in paediatric NB, concluded that SUVmax was not an independent prognostic predictor [34]. On the contrary, Li C et al. investigated the prognostic value of metabolic indices in paediatric NB, pointing that MTV and TLG were important prognostic predictors [35]. The prognostic value of traditional semi-quantitative metabolic parameters in NB patients remained controversial. Studies have noted that accurate assessment of tumour heterogeneity before treatment would help to improve patient prognosis [25, 36]. In our study, the intra-tumoural metabolic heterogeneity index was an independent predictor of prognosis. In contrast to traditional semi-quantitative metabolic parameters, the intra-tumoural metabolic heterogeneity index may be a more comprehensive and accurate approach to represent the higher heterogeneity of NB. We further grouped patients by using intra-tumoural metabolic heterogeneity index and BMI. Patients with high intra-tumoural metabolic heterogeneity and BMI would have a worse prognosis. Baseline heterogeneity could help clarify tumour characterization and improve therapy response for these patients.

Some limitations existed in our study. Firstly, our study was small and retrospective, and inherited a subjective selection bias. Secondly, all newly diagnosed NB patients were performed ^18^F-FDG PET/CT. Historically, ^18^F-FDG PET/CT was only performed for MIBG-negative tumours [37], while MIBG scans were not initially readily available in some regions. Thirdly, this study included different INSS stages patients, and therefore different treatment regimens were used that might have impacted outcomes. Fourthly, only the metabolic parameters of primary tumour were calculated, while the metabolic parameters of metastatic lesions might also have an impact on prognosis. Finally, partial volume effects may influence intra-tumoural metabolic heterogeneity, especially in patients with smaller primary lesions. Thus, a large multicentre prospective study should be conducted to confirm the present study results.

## Conclusion

In conclusion, our present study evaluated the value of ^18^F-FDG PET/CT intra-tumoural metabolic heterogeneity and semi-quantitative metabolic parameters in NB. Our results showed that intra-tumoural metabolic heterogeneity of the primary tumour was an independent prognostic factor for EFS. Furthermore, the combined predictive effect of intra-tumoural metabolic heterogeneity and BMI provided prognostic survival information in NB patients.

## Data Availability

The datasets of current study are available from the corresponding author on reasonable request.

## Abbreviations

SUVmean: the mean standardized uptake value
SUVmax: the maximum standard uptake value
SUVpeak: the peak standardized uptake value
MTV: metabolic tumour volume
TLG: total lesion glycolysis
AUC-CSH: areas under the curve of cumulative SUV-volume histogram
BMI: bone morrow involvement
MYCN: myelocytomatosis viral oncogene neuroblastoma derived homolog
EFS: event-free survival
COG: Children's Oncology Group
INSS: International Neuroblastoma Staging System
NSE: neuron-specific enolase
LDH: lactate dehydrogenase
PHOX2B: paired-like homebox 2B
ROC: receiver operating characteristics
